# Empiric Antibiotics in COVID 19: A Narrative Review

**DOI:** 10.7759/cureus.25596

**Published:** 2022-06-02

**Authors:** Elvina C Lingas

**Affiliations:** 1 Internal Medicine, Presbyterian Hospital, Albuquerque, USA

**Keywords:** antibiotic stewardship, empiric antibiotics, antibiotics, sars-cov2, covid19

## Abstract

Coronavirus disease 2019 (COVID-19) is a febrile respiratory illness caused by the severe acute respiratory syndrome coronavirus 2 (SARS-CoV-2). It may develop into respiratory failure or pneumonia. Antimicrobials have facilitated medical progress for decades. However, antimicrobial resistance (AMR) limits our ability to treat diseases and undermines efforts to attain health-related sustainable development and universal health coverage objectives. Antimicrobial resistance is a major concern that must be addressed immediately. The principles of appropriate prescription, optimal use of antimicrobials, quality diagnosis and treatment, and infection reduction and prevention have led to antimicrobial stewardship initiatives. During the current COVID-19 epidemic, there are possible hazards to antimicrobial stewardship measures and drug resistance. Many people with mild illnesses but without pneumonia or moderate infections with pneumonia are administered antibiotics. Antimicrobial therapy has no documented benefit in COVID-19 patients without microbial co-infection. COVID-19 patients may have an increased risk of developing concomitant microbial infections, which would necessitate antibiotic treatment. This review evaluated the role of empiric antibiotics in COVID-19 patients.

## Introduction and background

COVID-19 (coronavirus disease of 2019) continues to pose a threat to health systems around the world [1,2]. Antimicrobial resistance (AMR) had been a "hidden" epidemic affecting healthcare delivery around the world prior to the COVID-19 outbreak, claiming 700,000 deaths per year [3]. As stated by the World Health Organization (WHO), AMR occurs when pathogens such as parasites, fungi, bacteria, and viruses change and no longer respond to medication, increasing the difficulty of treating illnesses and the likelihood of negative consequences, mortality, and transmission of disease [4]. AMR was also identified by the WHO as one of the most serious risks to healthcare in 2019 [5]. The current COVID-19 outbreak may aggravate AMR, which is a global health concern [3]. The need to repurpose drugs to treat COVID-19 is growing, and the transfer of resources away from antimicrobial stewardship initiatives has resulted in the indiscriminate use of antibiotics in COVID-19 treatment, further complicating the problem [6]. COVID-19 currently governs many elements of global healthcare, including the responses of healthcare systems to antibiotic resistance. Even after the outbreak, the effects persisted. Antibiotic resistance is on the rise at an alarming rate, and only a few new antimicrobial drugs are on the horizon. It is critical to keep pace with pathogen epidemiology in order to make informed treatment decisions. Patients with COVID-19 are typically administered empiric antibiotic therapy for possible community-acquired bacterial pneumonia (CABP) since both the initial presenting symptoms of bacterial and viral infection are usually challenging to distinguish. However, the available evidence on coinfections in the COVID-19 setting does not support the routine use of empiric antibiotics for potential bacterial pneumonia [7-11]. Although COVID-19 patients had a low proportion of reported co-infections (8%), 72% received antimicrobial medication, according to a recent study [7]. Although it may be reasonable to start empiric antibiotics for CABP, antibiotic therapy should be reevaluated if COVID-19 pneumonia has been diagnosed. To reduce the risk of harmful effects, empirical antibiotics should not be prescribed unless the clinical presentation points towards severe bacterial pneumonia. Antibiotics should not be continued for longer than is necessary. Antibiotic use that is not based on evidence is a risk factor for resistance development [12], and this issue in resource-limited settings remains concerning in the case of COVID-19 because of lack of awareness, absence of antimicrobial stewardship committees, limited access to medicines, absence of both financial and human resources, ineffective antimicrobial stewardship, prescribers’ opposition, inefficient laboratory systems, poor infection prevention programs, and antibiotic policy, among others [6]. Despite the drawback of resistance selection, broad-spectrum antibiotics were shown to be the most commonly administered antibiotics in a prior study [13]. WHO has also warned against the use of antibiotics indiscriminately in COVID-19 treatment [14]. This current review evaluated the role of empiric antibiotics in COVID-19 patients.

## Review

Antibiotics and anti-microbial resistance in the COVID-19 era

Overuse of antibiotics has led to the development and spread of AMR, a serious health problem worldwide. One of the most significant strategies for resolving this problem is to ensure that the right antibiotic is administered at the right dose for the right amount of time and in a way that provides the optimal outcome while minimizing side effects and AMR. An antimicrobial stewardship program is known to possess these characteristics [15]. However, after the outbreak of COVID-19, there has been growing concern about the possibility of an increase in AMR as a result of increased antibiotic prescriptions for patients with COVID-19 [16].

According to various studies, antibiotics can safely and efficiently treat pneumonia that is recognized early in patients with COVID-19 and broad-spectrum antibiotics are routinely utilized [17-19]. According to a review based on data from 19 trials (2834 patients), the average antibiotic administration rate in COVID-19 is 74.0%, with merely 17.6% of patients with confirmed secondary infections [20]. Another study from South Africa found that bacterial co-infection with COVID-19 was uncommon when intensive care patients were admitted [21]. According to a meta-analysis, only 7.0% of patients hospitalized with COVID-19 developed a bacterial co-infection [22]. According to a recent multi-center investigation, only 86 (9.5%) of the 905 confirmed COVID-19 patients had bacterial co-infection [23]. This means that only a limited number of patients with COVID-19 require antibiotics to treat bacterial pneumonia or other co-infections [24].

The identification of probable bacterial co-infection in critically ill and hospitalized patients is challenging [25]. Increased use of broad-spectrum antibiotics will not only render the medications ineffective but will also result in the emergence of extremely drug-resistant bacteria. This poses a significant risk for antimicrobial stewardship. In several African countries, for example, the use of azithromycin has increased as a result of the pandemic [26,27]. Azithromycin use in the community to reduce the likelihood of hospitalization in suspected COVID 19 patients proved to be unsuccessful [28,29]. In conclusion, antibiotics should be used with caution and withheld unless the patient's true need has been established. While a lack of access to antibiotics can be deadly, it is critical to ensure that these life-saving drugs are maintained and utilized with extreme caution [25].

Bacterial Co-infection and COVID-19

Despite the fact that only 1% of people have a proven bacterial co-infection, according to a study conducted by a Chinese adult infectious disease center, 71% of hospitalized COVID-19 patients receive antibiotics [30]. Another study performed at two Chinese hospitals reported that out of 95% of patients who received antibiotics, only 15% of patients with COVID-19 had bacterial co-infection [31]. A 2020 study in the UK showed that 3.2% of hospitalized COVID-19 patients showed positive blood cultures within the first five days of admission and the rate increased to 6.1% after that. In 34.8% of cases, microbe was confirmed in the sputum sample [9].

Early reports from Wuhan, China, affected the initial trend of antibiotic administration in COVID-19 patients. A retrospective cohort study showed that 50% of COVID-19 patients who died had bacterial co-infection [31]. This is similar to findings from a study that looked into the 1918 influenza pandemic post-mortem cultures, which showed that the majority of casualties died from bacterial pneumonia [32]. Haemophilus influenza, Pseudomonas aeruginosa, and Mycoplasma pneumoniae were the microorganisms most frequently recovered from induced bronchoalveolar lavage in patients with COVID-19 [22]. In the face of this growing pandemic, the antibiotic approach should be administered based on existing guidelines and insights learned from prior viral outbreaks. For example, the term "super-spreader" has been applied to situations such as hospitals, cruise ships, and other events involving large population movements and high transmission rates [33]. Bacterial co-infections in hospitalized patients have been found to be super-spreaders of resistant germs in previous SARS-CoV epidemics, with each individual potentially infecting more than ten more people [34]. It has been shown that in COVID-19, 80% of the spread is linked to 20% of patients. It's unclear whether SARS-CoV-2 is linked to the same pattern of resistant bacterial spread as SARS-CoV [33]. However, to reduce the danger of hospital transmission and AMR infections, this is an important factor to remember, particularly in low- and middle-income countries (LMICs) where infection control and prevention measures in hospitals are typically ineffective [35].

Antibiotic Selection Challenges for COVID-19 Patients

Antibiotics should be used to treat nosocomial bacterial co-infections if the risk of requiring invasive mechanical ventilation justifies it [36]. In LMICs, the shortage of ventilators decreases this risk, resulting in a significant reduction in unnecessary antibiotic use [37]. On the other hand, short-term peripheral venous catheters have been linked to LMICs that have higher rates than in high-income countries (HICs) [38]. There is a chance that the rising number of COVID-19-infected hospital patients will lead to nosocomial catheter-associated infections, and antibiotics will be used more frequently. Antimicrobial use, infection control, and prevention technologies could help mitigate this risk [39]. Previous studies have shown that there are variances between HIC and LMIC for the same bacterial infections in terms of populations at risk, antibiotic susceptibility, pathogen distribution frequency, and clinical manifestations. Studies on bacterial coinfections in patients with COVID-19 in LMICs may be interesting [40]. In reality, the threat of COVID-19 may present an opportunity for LMICs to adopt antimicrobial stewardship programs in accordance with WHO standards, such as antimicrobial stewardship team education and training, resistance surveillance, antibiotic use surveillance, and clinical guidelines development [41].

Bacterial Identification Challenges in Pneumonia Patients

Antibiotics are often prescribed empirically in COVID-19 patients, as they are in most illnesses [42]. Only 28% of patients with COVID-19 had enough sputum to develop a Gram stain, despite the fact that 98% of patients exhibited bilateral lung involvement on chest X-rays [42]. The fact that pathogens can only be identified from sputum samples in patients with pneumonia is further hampered by the fact that only 23% of cases provide results [43]. Furthermore, concerns about aerosol-generating processes limit the capacity to get adequate sputum samples for the identification of bacteria and other microbiological techniques [44]. However, if appropriate personal safety equipment is readily available, crucial diagnostics should be reconsidered [45].

Probiotics as a Treatment for Antibiotic-Related Complications

For more than 50 years, probiotics have been examined for a number of ailments, including allergy prevention, cancer and gastrointestinal disease therapy, and the avoidance of a variety of gastrointestinal problems. In cases when the typically protective microflora has been disrupted, probiotics are the most beneficial [46-49]. Living microorganisms (bacteria or yeast) with one or more different mechanisms of action are known as probiotics, such as toxin degradation, barrier strengthening, pathogen attachment inhibition, immunological control, or trophic effects [50,51]. Several probiotics have been found to reduce the risk of AAD (antibiotics-associated diarrhea) and CDI (*Clostridium difficile* infections), but few studies have been published on their ability to modulate the immune response [52-54].

Antibiotic-associated complications prevention in patients with COVID-19

Antibiotic-Associated Diarrhea

AAD is a type of diarrhea that is induced by antibiotic exposure. This can happen while taking antibiotics or up to eight weeks after they have ceased working [55]. Despite the fact that the etiologies of AAD are diverse and not all infections are now identified, *C. difficile* is responsible for almost one-third of AAD cases. AAD affects about 20% of patients, but the rate varies based on comorbidities, antibiotic type, hospitalization, age, and other risk factors [55]. Extended hospital stays, greater mortality rates, and higher healthcare expenses have all been linked to AAD [55]. Unfortunately, few studies have reported the frequency of AAD in published reports addressing the clinical courses of COVID-19 patients. This is a well-known side effect of antibiotic exposure; therefore, it should be considered when managing COVID-19 patients.

C. Difficile Infections

The most common cause of healthcare-associated gastrointestinal illnesses is *C. difficile*. In 2017, 460,000 cases were reported, with a 5.2% fatality rate attributed to the disease and anticipated medical costs of USD 5 billion [56,57]. The association between COVID-19 and CDI is still under investigation. At Detroit Medical Center Hospitals, Sandhu et al. discovered nine cases of CDI in COVID-19 patients [58]. Two patients had diarrhea and were positive for *C. difficile* at the time of admission; three had previously had CDI; and seven developed CDI after receiving their COVID-19 diagnosis, which took a median of six days. All of these individuals had been on antibiotics for an average of five days prior to the discovery of CDI. Four patients died, and one was assigned to hospice care, with a median age of 75 years, and 78% of the patients were female. While patients' mortality and prognosis were most likely influenced by their SARS-CoV-2 infection, they also had a number of other CDI risk factors, such as hospitalization, older age, and antibiotic exposure. A recent case report from Spain highlighted the potential for additional morbidity associated with CDI. A 64-year-old woman had severe colitis caused by *C. difficile* following a one-month hospital admission for COVID-19 infection with bilateral pneumonia, necessitating colectomy ten days later [59]. Bentivegna et al. also performed a retrospective case-control study in eight Italian hospitals, comparing COVID-19 patients with and without CDI [60]. COVID-19 patients with CDI had more COVID-19-related complications than COVID-19 patients without CDI and spent more time in the hospital (on average, 16 days longer). At a hospital in Italy, CDI was discovered in 5% of patients with COVID-19 and 3% of non-COVID-19 patients, according to Bentivegna et al., who tested 483 inpatients [60]. In a study of 4973 patients at two New York hospitals, Laszkowska et al. discovered slightly fewer CDI cases (5%) in patients with COVID-19 than in COVID19 free individuals [61]. Despite the increased antibiotic use in COVID 19 patients, recent research has indicated an unexpected decline in CDI infections. Following the conversion of their hospital in Mexico City to a COVID-19-only facility, Ochoa-Hein et al. identified a considerable decline in the number of CDI cases compared to the previous year (9.3/10,000 patient-days) [62]. Hazel et al. reported a lower rate of CDI (2.15/10,000 bed-days) during the pandemic than that in the previous year (4.24/10,000 bed-days) at their hospital in Ireland, which they attributed to COVID-19 patients' lower bed occupancy and enhanced infection control procedures [63]. After establishing a multimodal infection management approach for patients with COVID-19, Wee et al. reported no significant increases in CDI [64]. Improved infection control measures focused on preventing the spread of SARS-CoV-2 could also help reduce the spread of other healthcare-associated infections such as *C. difficile*. Other factors, such as lower hospital bed occupancy and lower *C. difficile* incidence, may also play a role in the lower reported rates of CDI. Even when stronger infection control programs are implemented, vigilance is required for future outbreaks in the midst of the epidemic.

## Conclusions

Despite a low proportion of confirmed community-onset bacterial co-infections, most patients hospitalized with COVID 19 received early empiric antibiotics. Larger studies are necessary to establish guidelines for the benefits of early empiric antibiotics. It is imperative to implement antibiotic stewardship programs in hospitals and to review the need for continuous antibiotic use to prevent complications from antibiotic use such as *C. difficile* infections.
